# Evaluating the Impact of Patellar Resurfacing in Medial Stabilized Total Knee Arthroplasty: A Retrospective Cohort Study

**DOI:** 10.1111/os.70371

**Published:** 2026-07-03

**Authors:** Isaac Sontag‐Milobsky, Giancarlo Riccobono, T. Jacob Selph, Kevin D. Hardt, David W. Manning

**Affiliations:** ^1^ Loyola University Chicago Stritch School of Medicine Maywood Illinois USA; ^2^ Northwestern University Feinberg School of Medicine Chicago Illinois USA; ^3^ Department of Orthopaedic Surgery Northwestern University Feinberg School of Medicine Chicago Illinois USA

**Keywords:** arthroplasty, articular, knee, osteoarthritis, patella, patient reported outcome measures, range of motion, replacement

## Abstract

**Objectives:**

Patellar resurfacing in total knee arthroplasty (TKA) remains a debated topic. Medial stabilized (MS) implants have gained popularity in the United States in recent years as they more closely mimic native femoral‐tibial and patella‐femoral knee kinematics. However, little is known about the effects of patellar resurfacing with a specific round‐on‐round, one‐to‐one congruent, medial stabilized implant. The purpose of this study was to evaluate whether patellar resurfacing influences pain, function, patient‐reported outcomes, and perioperative outcomes in patients undergoing primary MS TKA with this implant.

**Methods:**

A retrospective cohort study was conducted on 107 patients undergoing primary MS TKA with the Sphere implant (Medacta International, Castel San Pietro, SZ). Patients were divided into resurfacing (R, *n* = 51) and non‐resurfacing (NR, *n* = 56) groups. Outcomes included pain scores, range of motion (ROM), patient‐reported outcome measures (PROMs), and perioperative variables. PROs collected included PROMIS Physical Function and Pain Interference CATs, Knee Society Score (KSS), Visual Analog Scale (VAS) pain score (0–100), Knee injury and Osteoarthritis Outcomes Score (KOOS), Forgotten Joint Score (FJS), patellofemoral pain questionnaire, and the Samsung Medical Center (SMC) patellofemoral scoring system. Comparative statistical analyses were performed using chi‐square tests, *t*‐tests, and Mann–Whitney U tests.

**Results:**

No significant differences were found in postoperative pain scores (VAS), opioid consumption, or overall PROMs between groups. However, the non‐resurfacing group demonstrated greater ROM at 12 months (121.5° vs. 116.4°, *p* = 0.03) and higher KOOS ADL and symptom subscales. Resurfacing was associated with increased operative time (*p* < 0.001) and tourniquet time (*p* = 0.03); hospital LOS was shorter in the resurfacing group by six hours (*p* < 0.05).

**Conclusion:**

In MS TKA, patellar resurfacing did not confer significant advantages in pain relief or functional outcomes at one year. These findings support an individualized approach to patellar resurfacing in MS TKA as each technique is deemed non‐inferior.

## Graphical abstract

In medial‐stabilized total knee arthroplasty with a single implant design, patellar resurfacing did not improve pain or patient‐reported outcomes and slightly increased operative time, while non‐resurfaced knees showed marginally better range of motion and function, supporting an individualized, non‐inferior approach to patellar management.

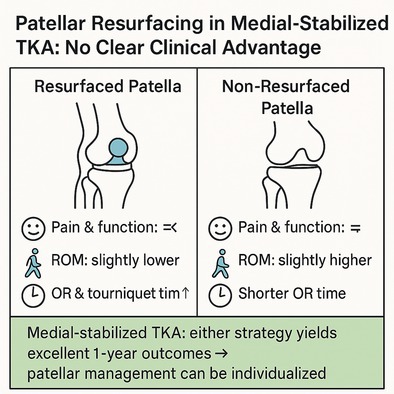

## 1 Introduction

Total knee arthroplasty (TKA) is a highly effective treatment for end‐stage knee osteoarthritis, with procedure volumes continuing to rise [1, 2, 3, 4]. Despite excellent overall outcomes, patellar management remains one of the most debated aspects of primary TKA [5, 6, 7]. Large meta‐analyses in contemporary posterior cruciate–retaining and posterior cruciate–substituting designs suggest patellar resurfacing may reduce anterior knee pain and some reoperations, although differences in patient‐reported outcome measures (PROMs) are often small or inconsistent [8, 9, 10]. As a result, resurfacing practices remain heterogeneous across surgeons and regions.

Medial‐stabilized (MS) TKA systems have gained popularity because their design aims to better reproduce native femorotibial and patellofemoral kinematics and improve mid‐flexion stability [11]. Registry data demonstrate rapidly increasing adoption of MS constructs in recent years [12]. However, whether the benefits of patellar resurfacing observed in other implant classes translate to MS designs is unclear. Recent studies evaluating MS and other “patellar‐friendly” designs have reported minimal differences in PROMs, anterior knee pain, or reoperation rates between resurfaced and non‐resurfaced patellae [13, 14]. Importantly, MS systems are not uniform, and implant‐specific geometry may influence patellofemoral tracking, contact mechanics, and clinical outcomes. To date, limited evidence addresses patellar resurfacing specifically in the setting of a round‐on‐round, one‐to‐one congruent MS implant (Sphere; Medacta International, Castel San Pietro, Switzerland).

Accordingly, the purpose of this study was to evaluate whether patellar resurfacing influences pain, function, PROMs, and perioperative outcomes in patients undergoing primary MS TKA with the Sphere implant. We hypothesized that patellar resurfacing would be associated with improved pain control and superior patient‐reported outcomes compared with non‐resurfacing.

## 2 Methods

### 2.1 Study Design

This is a retrospective cohort study designed to evaluate the impact of patellar resurfacing on pain and functional outcomes in patients undergoing total knee arthroplasty with a single medial stabilized TKA implant. The study was conducted through a single tertiary care center and included patients who underwent primary TKA with a single medial stabilized system between January 2018 and September of 2023. This study was conducted in accordance with the ethical standards of the Declaration of Helsinki and was reviewed and approved by our Institutional Review Board (IRB No. [STU00209310]). Written informed consent was obtained from all patients prior to participation.

### 2.2 Patient Selection

Inclusion criteria for participation were age 18 years or older, diagnosed with osteoarthritis of the knee, and underwent TKA with a single medial stabilized system. The severity of patellofemoral arthritis was not used as an inclusion criterion; instead, all eligible patients with knee osteoarthritis were included regardless of patellofemoral arthritis grade, and baseline patellofemoral arthritis severity was subsequently characterized radiographically and analyzed as a covariate. All included patients had complete operative records and a minimum of 12 months of clinical and patient‐reported outcome follow‐up, with the 1‐year postoperative visit serving as the primary follow‐up time point. Exclusion criteria were inflammatory arthropathies, trauma, patients who underwent radiofrequency ablation of the genicular nerves, patella less than 12 mm, and knee with valgus deformities.

Patients were assigned to study groups according to whether the patella was resurfaced at the time of primary TKA, yielding a resurfacing group and a non‐resurfacing group. Patellar management was determined by the operating surgeon rather than by patient‐ or knee‐specific indications: one surgeon resurfaced the patella in all primary TKAs, while the other did not resurface the patella in any primary TKA. Accordingly, resurfacing was not selectively applied on the basis of patellofemoral arthritis severity, anterior knee pain, patellar size, or intraoperative tracking, and group membership was determined solely by surgeon assignment.

### 2.3 Patient Allocation and Surgeon Assignment

Patellar management was surgeon‐dependent: one surgeon performs patellar resurfacing in all primary TKAs, whereas the other does not resurface the patella in any primary TKA. Patients were not assigned to surgeons based on patellar management strategy. Surgeon assignment occurred through standard clinical scheduling and referral processes, and we did not identify systematic factors (e.g., referral pathways, clinic schedules, or patient preferences for patellar resurfacing) that influenced allocation to either surgeon during the study period. Consequently, treatment group membership was fully determined by the operating surgeon.

### 2.4 Data Collection

A list of all patients who had undergone primary TKA and who were at least 1 year postoperative was obtained from our institution's Enterprise Data Warehouse (EDW). Eligible patients were then contacted by a member of the research team and scheduled to come in for a 1‐year postoperative examination. This included a detailed physical exam and obtaining anterior–posterior, lateral, and sunrise radiographs. Functional outcomes were assessed using the Patient‐Reported Outcomes Measurement Information System (PROMIS) Physical Function and Pain Interference computerized adaptive testing, Knee Society Score, Visual Analog Scale (VAS) pain score (0–100), patellofemoral pain questionnaire, Knee Injury and Osteoarthritis outcome Score (KOOS), Forgotten Joint Score (FJS), and the Samsung Medical Center (SMC) patellofemoral scoring system questionnaire.

The outcome measures were defined and scored as follows. The PROMIS Physical Function and Pain Interference computerized adaptive tests are reported as T‐scores normalized to the United States general population (mean 50, standard deviation 10), with higher Physical Function scores indicating better function and higher Pain Interference scores indicating greater pain‐related interference. The Knee Society Score (KSS) comprises a knee score and a function score, each ranging from 0 to 100, with higher values reflecting better knee status and function. The Visual Analog Scale (VAS) pain score ranges from 0 (no pain) to 100 (worst imaginable pain). The Knee Injury and Osteoarthritis Outcome Score (KOOS) yields five subscales (pain, symptoms, activities of daily living, sport and recreation, and quality of life), each scored from 0 to 100, with higher scores indicating better outcomes. The Forgotten Joint Score (FJS) ranges from 0 to 100, with higher scores indicating greater ability to forget the artificial joint in daily life. The Samsung Medical Center (SMC) patellofemoral scoring system evaluates patellofemoral‐specific pain and function, with higher scores reflecting greater patellofemoral symptom burden. All patient‐reported outcome measures were collected at the 1‐year postoperative visit, and between‐group differences were evaluated using the comparative statistical methods described below. Data was then collected from the electronic medical record, with the primary exposure variable being did the patient undergo patellar resurfacing during TKA (resurfacing group) or not (non‐resurfacing group). Demographic data including age, gender, BMI, race, ethnicity, and comorbidities was also collected via the EMR, as well as surgical data including operative time, total blood loss, and total tourniquet time. Hospital data which included hemoglobin delta between pre‐op and morning after surgery, discharge hemoglobin, length of stay, and opioid equivalent use during the hospital stay.

### 2.5 Radiographic Assessment of Patellofemoral Arthritis Severity

Preoperative patellofemoral arthritis severity was assessed using available Merchant radiographs. Three independent reviewers graded patellofemoral arthritis severity using the Merchant classification system. Reviewers were blinded to postoperative patient‐reported outcomes. Interrater reliability was assessed using quadratic weighted Cohen's kappa. Patient‐level Merchant grade was determined using the median grade across reviewers for descriptive analyses. Merchant grade distributions were compared between resurfaced and non‐resurfaced cohorts, and grades were further categorized as mild‐to‐moderate disease (Merchant 0–2) or severe disease (Merchant 3–4) for subgroup comparison.

### 2.6 Surgical Methods

All procedures in this study were performed using a one‐to‐one congruent, medial ball and socket, medially stabilized TKA system, ensuring consistency across both patella‐resurfaced and non‐resurfaced groups. Two experienced surgeons conducted the procedures, with one exclusively operating on the patella‐resurfaced group and the other on the non‐resurfaced group, which included denervation and partial lateral facetectomy. This approach minimized intra‐group variability in technique and allowed for a more controlled comparison between the two groups. Under spinal anesthesia, patients were positioned supine with a tourniquet applied to the operative leg. A midvastus approach was used to expose the knee joint and all necessary bony cuts and ligament balancing were performed according to traditional mechanical alignment principles. After cementing the components, the tourniquet was deflated, and electrocautery was used to achieve hemostasis. All patients received preoperative and post‐tourniquet‐deflation intravenous Tranexamic Acid [15]. This standardized surgical method aimed to maintain uniformity across cases and optimize postoperative outcomes in both study groups.

### 2.7 Statistical Methods

Descriptive statistics, comparative data analyses and figure generation were performed via Python 3.12.2 (Python Software Foundation, Wilmington, DE). Categorical data were compared via Chi‐Square (χ^2^) analysis or Fisher's exact test. Numeric data were analyzed via Shapiro–Wilk and Levene's tests to determine the appropriateness of comparative analyses via either *t*‐testing or Mann–Whitney U testing, and the appropriate test was applied. Effect sizes for significant differences were quantified via Cohen's d for *t*‐testing and rank‐biserial correlation (*r*) for Mann–Whitney U test. A priori power analysis with 1—*β* = 0.80 suggested a minimum sample size of 102 patients. Significance level for all analyses was set at *α* = 0.05. Multiple outcomes were evaluated, including several patient‐reported outcome measures and subscales. Given the exploratory, hypothesis‐generating nature of these secondary comparisons, we did not apply formal adjustment for multiple testing. Therefore, *p*‐values for secondary outcomes should be interpreted cautiously as descriptive measures of statistical compatibility rather than definitive evidence of effect. Between‐group differences in baseline patellofemoral arthritis severity were assessed using the Mann–Whitney U test. A chi‐square test was used to compare the distribution of mild‐to‐moderate versus severe patellofemoral arthritis between cohorts. Patient‐level ordinal logistic regression was additionally performed to evaluate whether the resurfacing cohort was associated with Merchant grade. Spearman correlation analyses were performed within each cohort to assess associations between baseline Merchant grade and postoperative PROMs.

## 3 Results

### 3.1 Patient Characteristics

A total of 107 total knee arthroplasty patients were enrolled in the study. 51 patients underwent patellar resurfacing (R) and 56 did not (NR). Key patient demographic data such as age, sex, race, and ethnicity are detailed in Table 1. There was no significant difference between sample groups for demographic data analyzed.

**TABLE 1:** Baseline demographics.

Variable	Patellar resurfacing	Non‐resurfacing	*p*‐value
Number of knees	51	56	
Age (Mean ± SE, years)	69 ± 1.7	68 ± 1.2	0.631^a^
BMI (Mean ± SE, kg/m^2^)	31.95 ± 0.91	30.13 ± 0.68	0.393^a^
Sex (Female: Male)	32:19	39:17	0.540^b^
Race (Asian: Black:White)	1:8:40	2:9:45	0.896^c^
Ethnicity (Hispanic: Non‐Hispanic)	1:48	3:52	0.620^b^

*Note:* Patients undergoing medial pivot primary total knee arthroplasty (*N* = 107) with and without patellar resurfacing.

^a^ Mann–Whitney U test.

^b^ Chi‐squared test.

^c^ Fisher's exact test.

Preoperative Merchant radiographs were available and gradable for 95 patients, including 46 patients in the non‐resurfacing cohort and 49 patients in the resurfacing cohort. Interrater reliability for Merchant grading demonstrated substantial agreement, with pairwise quadratic weighted Cohen's kappa values ranging from 0.63 to 0.80. Baseline radiographic patellofemoral arthritis severity was not significantly different between cohorts (Table 2). Mean Merchant grade was 2.21 in the resurfacing cohort and 1.82 in the non‐resurfacing cohort (*p* = 0.075). Ordinal logistic regression similarly demonstrated no statistically significant association between treatment cohort and Merchant grade (OR 1.93, 95% CI 0.94–3.96, *p* = 0.072). When Merchant grades were categorized as mild‐to‐moderate (Merchant 0–2) or severe (Merchant 3–4), severe patellofemoral arthritis was present in 15 patients (30.6%) in the resurfacing cohort and 8 patients (17.4%) in the non‐resurfacing cohort, which was not statistically significant (*p* = 0.211, Table 3). Because all eligible patients with knee osteoarthritis were included regardless of patellofemoral arthritis severity and group assignment was determined solely by the operating surgeon rather than by patellofemoral arthritis grade, the numerically higher proportion of severe patellofemoral arthritis in the resurfacing cohort reflects the distribution of disease within each surgeon's underlying patient population rather than selective inclusion or grouping. The absence of a statistically significant between‐group difference in Merchant grade indicates that baseline patellofemoral arthritis severity was reasonably balanced across cohorts.

**TABLE 2:** Baseline merchant classification by patellar resurfacing status.

Median merchant grade	Non‐resurfaced (*n* = 46)	Resurfaced (*n* = 49)
0	6	2
1	14	11
2	18	21
3	8	15
4	0	0

*Note:* Merchant grade was determined using the median grade across three independent reviewers. Pairwise quadratic weighted Cohen's kappa values ranged from 0.63 to 0.80, indicating substantial interrater agreement. Mean Merchant grade was 2.21 in the resurfacing cohort and 1.82 in the non‐resurfacing cohort (Mann–Whitney U test, *p* = 0.075). Patient‐level ordinal logistic regression demonstrated no statistically significant association between resurfacing cohort and Merchant grade (OR 1.93, 95% CI 0.94–3.96, *p* = 0.072).

**TABLE 3:** Mild‐to‐moderate vs. severe patellofemoral arthritis by resurfacing status.

PF arthritis category	Non‐resurfaced (*n* = 46)	Resurfaced (*n* = 49)	*p*‐value
Merchant 0–2	38 (82.6%)	34 (69.4%)	
Merchant 3–4	8 (17.4%)	15 (30.6%)	0.211

*Note:* Merchant grades 0–2 were categorized as mild‐to‐moderate patellofemoral arthritis, while Merchant grades 3–4 were categorized as severe patellofemoral arthritis. Groups were compared using chi‐square analysis.

### 3.2 Short‐Term Outcomes

Intra‐operative tourniquet time (102.4 ± 11.2 R, 96.7 ± 16.1 NR, *p* = 0.03, *r* = 0.26) and total operative time (144.8 ± 31.8 R, 120.0 ± 29.1 NR, *p* = 5.3e^−5^, d = 0.82) were significantly longer for patients whose TKA included patellar resurfacing. There was no significant difference between cohorts for post‐operative morphine milligram equivalents needed for adequate pain control (70.6 ± 53.7 R, 67.6 ± 79.1 NR, *p* = 0.25). Length of stay (LOS) differed significantly between cohorts, with patients in the resurfacing group demonstrating a longer hospital stay than those in the non‐resurfacing group (44.2 h vs. 40.1 h; *p* = 0.024).

**FIGURE 1:**
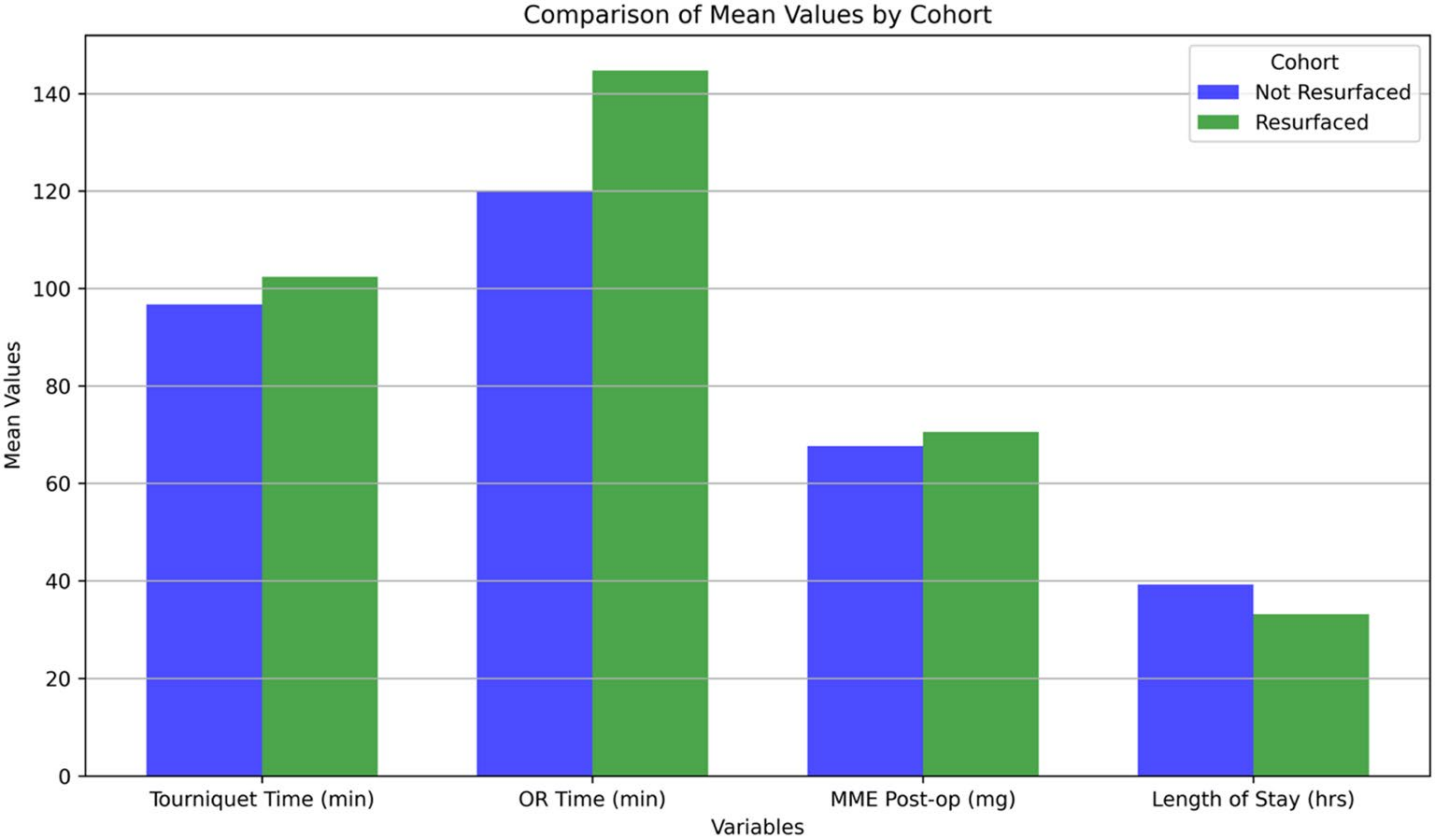
Surgical characteristics and pain control via pharmacotherapy. The bar chart illustrates the mean values for tourniquet time, operating room (OR) time, length of stay, and postoperative morphine milligram equivalents (MME) between the patellar resurfaced and non‐resurfaced groups.

**FIGURE 2:**
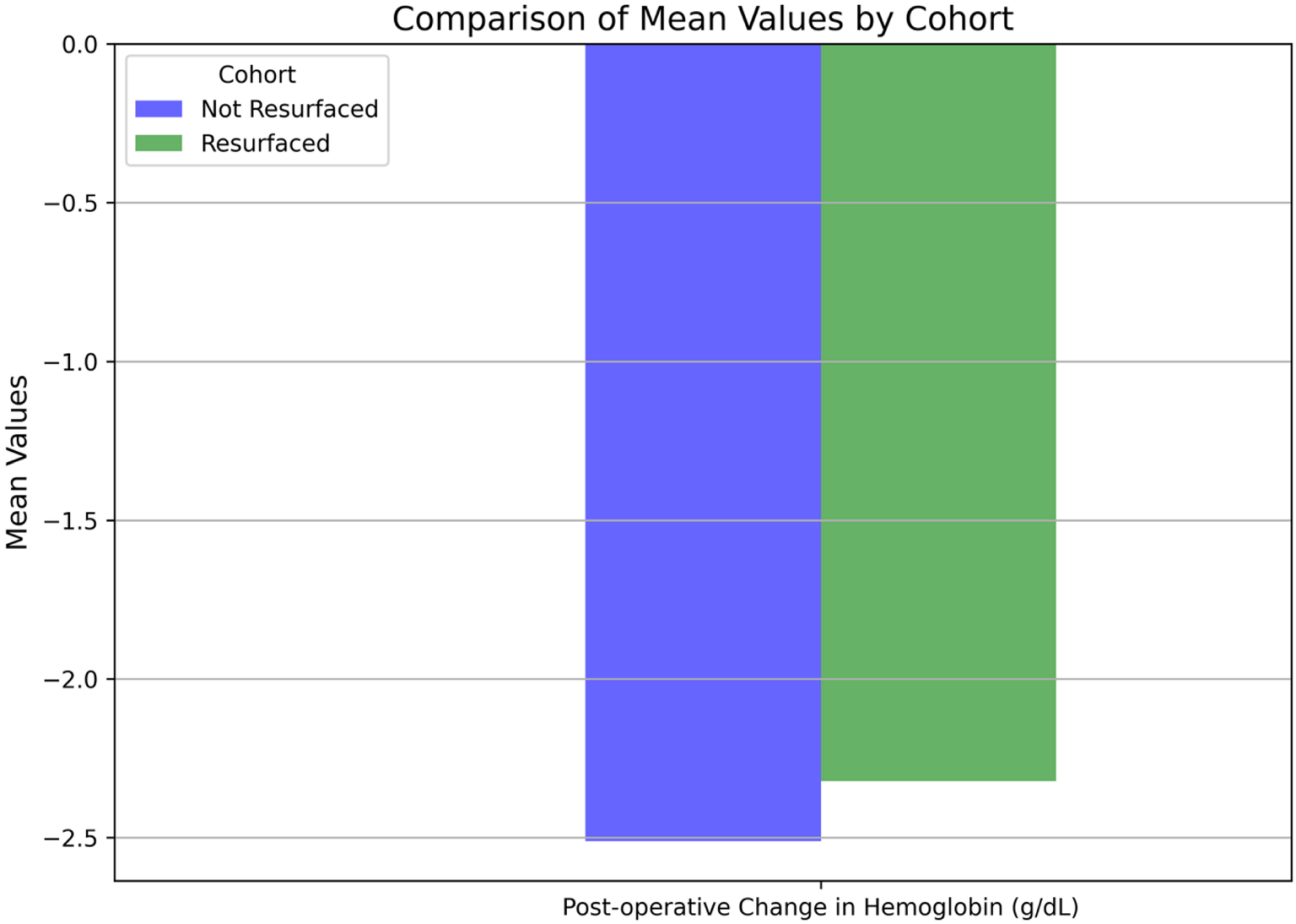
Hemoglobin delta. The vertical bar chart depicts the average difference in hemoglobin (g/dL) between pre‐op and the morning after surgery.

Tourniquet time, OR time, LOS, and post‐operative MME data are visualized in Figure 1. Post‐operative change in hemoglobin did not change significantly by resurfacing status (−2.3 ± 1.1 R, −2.5 ± 0.8 NR, *p* = 0.61). Post‐operative ∆hemoglobin data are visualized in Figure 2.

### 3.3 12‐Month Outcomes

**FIGURE 3:**
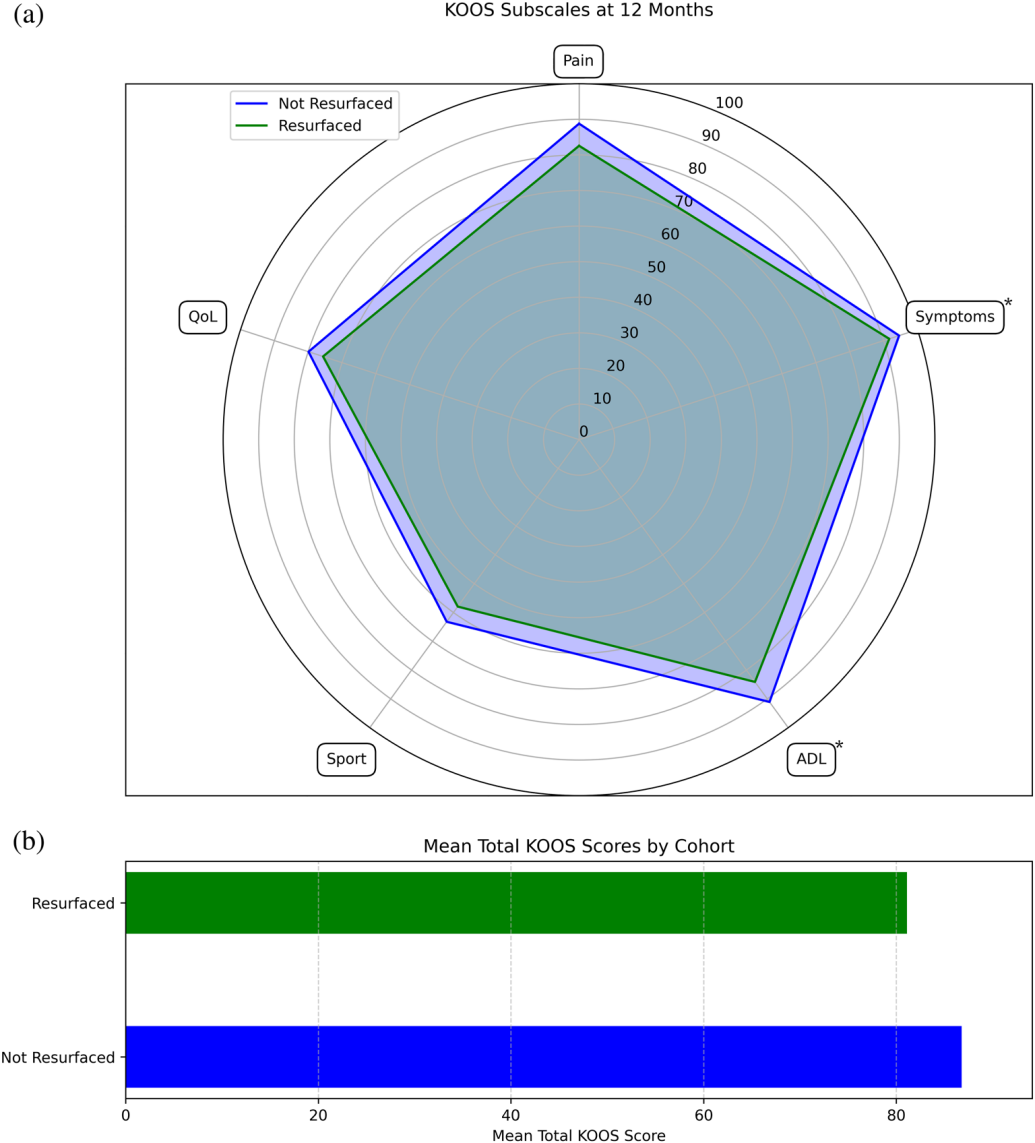
Knee injury and osteoarthritis outcome score (KOOS). The radar chart (a) portrays the mean values for each of the five KOOS subscales (pain, symptoms, activities of daily living [ADL], sport, quality of life [QoL]). The horizontal bar chart (b) represents the average total KOOS for both groups. Data from both charts is at 12 months post‐op. * Statistically significant difference.

**FIGURE 4:**
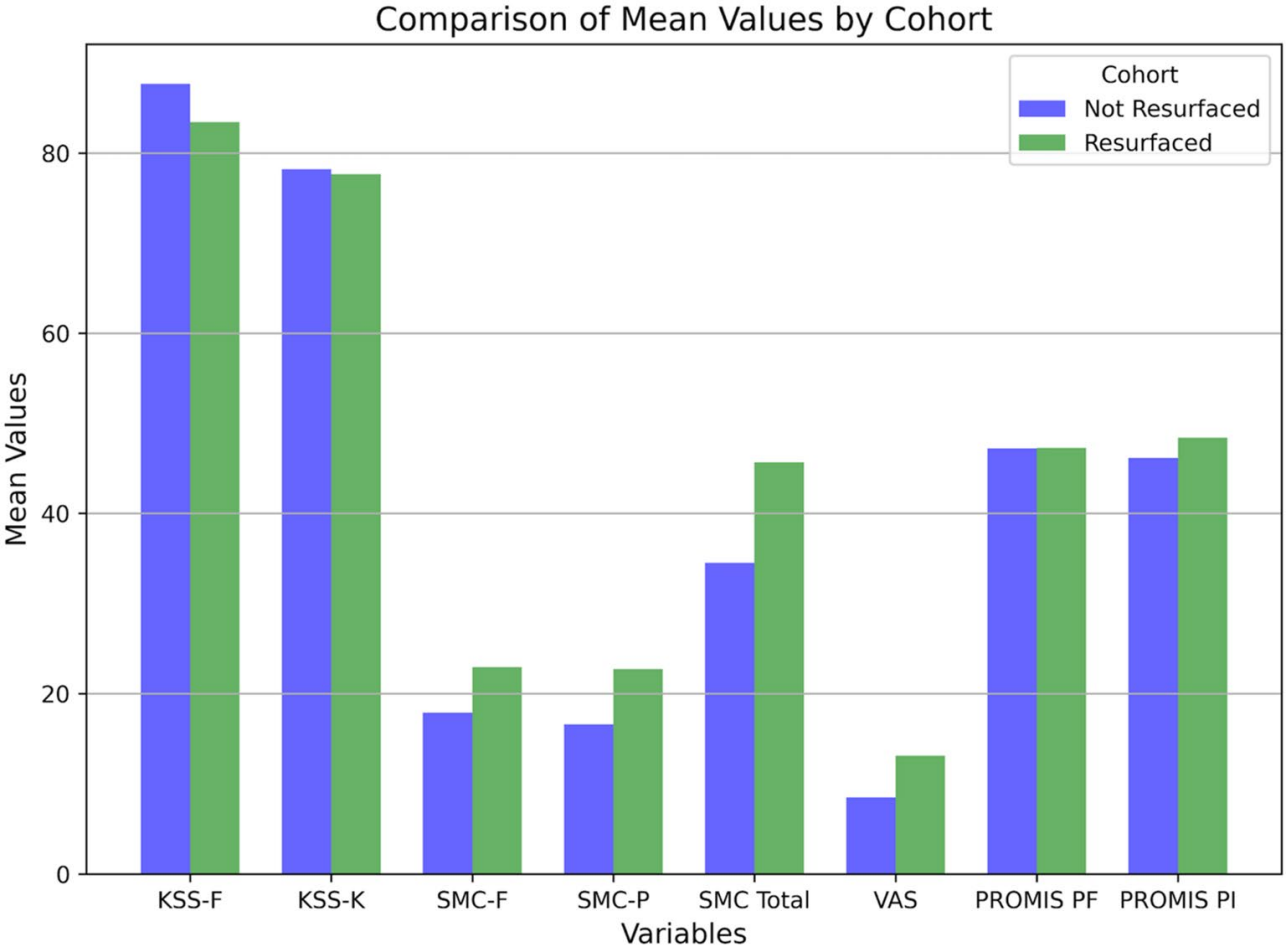
Patient‐reported outcomes (PROs). The bar chart illustrates the mean values for the following PROs at 12 months post‐op: knee society score (KSS, F‐function and K‐knee), samsung medical center (SMC, F‐function and P‐pain) patellofemoral score, visual analog score (VAS), and patient‐reported outcomes measurement information system (PROMIS, PF‐physical function and PI‐pain interference). * Statistically significant difference.

**FIGURE 5:**
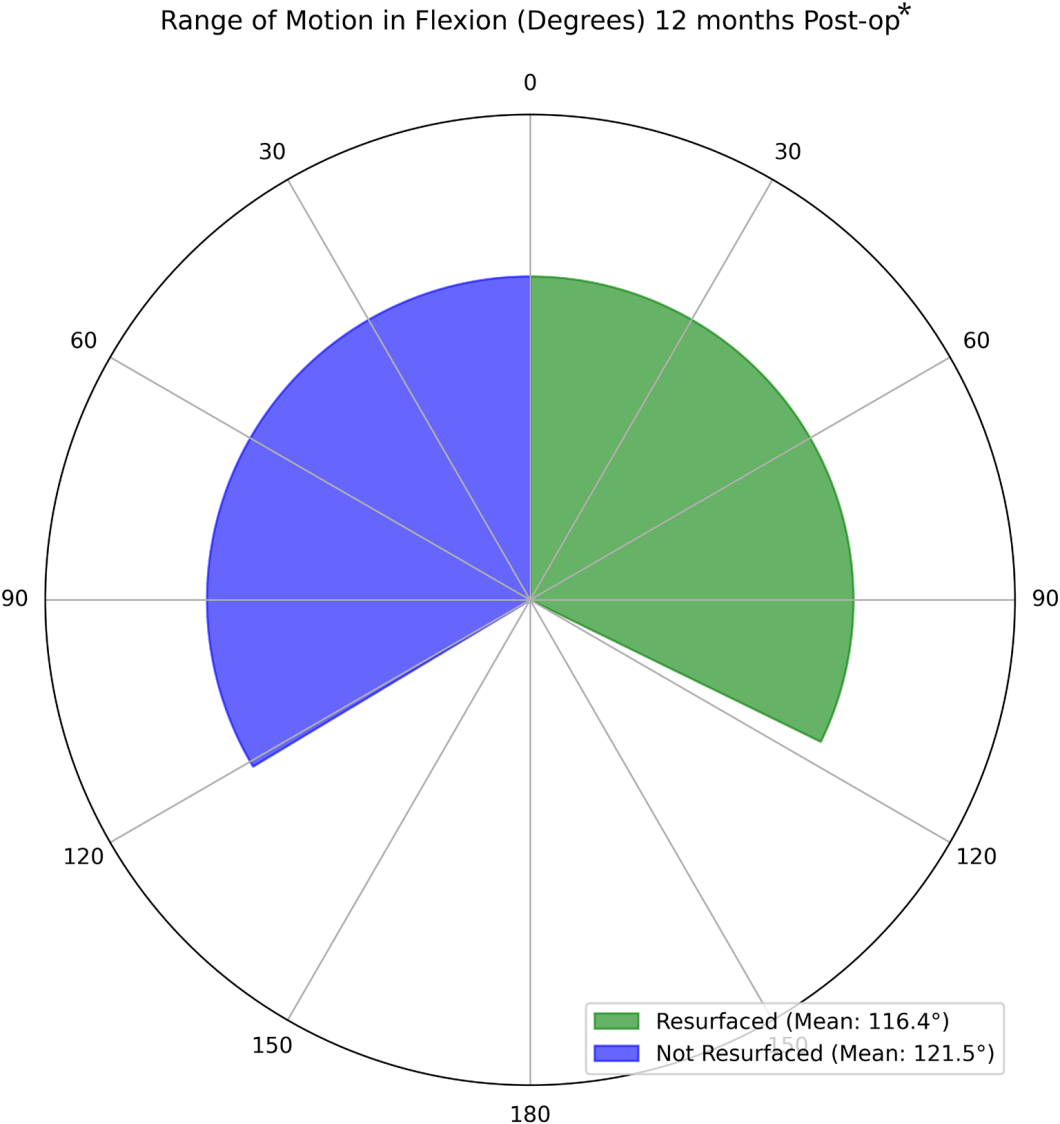
Range of motion (ROM). The polar bar chart illustrates the mean degrees of ROM achieved in both groups at 12 months post‐op. * Statistically significant difference.

Average KOOS ADL (84.1 ± 18.4 R, 91.1 ± 11.9 NR, *p* = 0.04, *r* = −0.23) and symptom (91.7 ± 7.3 R, 94.6 ± 5.3 NR, *p* = 0.04, *r* = −0.23) subscales were significantly higher for patients without resurfaced patellae. Average KOOS pain (82.6 ± 19.9 R, 88.8 ± 12.3 NR, *p* = 0.12), quality of life (75.6 ± 19.2 R, 79.9 ± 16.4 NR, *p* = 0.25), and sport (57.9 ± 30.2 R, 63.2 ± 29.2 NR, *p* = 0.36) subscales were not significantly different between cohorts. Average total KOOS scores (81.1 ± 16.5 R, 86.8 ± 10.9 NR, *p* = 0.11) were not significantly different between cohorts. KOOS data are visualized in Figure 3a,b. Average KSS knee scores were not significantly different between cohorts (78.2 ± 10.6 NR, 77.7 ± 10.5 R, *p* = 0.68). Average scores were not significantly different between cohorts for KSS function (87.7 ± 16.2 NR, 83.4 ± 19.5 R, *p* = 0.23). SMC function (22.9 ± 20.9 R, 17.9 ± 18.3 NR, *p* = 0.21), SMC pain (22.7 ± 22.1 R, 16.6 ± 17.6 NR, *p* = 0.30), SMC total (45.7 ± 41.0 R, 34.5 ± 34.9 NR, *p* = 0.19), VAS (13.1 ± 18.5 R, 8.5 ± 14.4 NR, *p* = 0.21), PROMIS PF (47.3 ± 6.9 R, 47.2 ± 5.6 NR, *p* = 0.97), or PROMIS PI (48.4 ± 8.0 R, 46.2 ± 7.4 NR, *p* = 0.16). KSS, SMC, VAS, and PROMIS data are visualized in Figure 4. Physical examination at the time of 12‐month data collection revealed significantly larger range of motion in flexion for patients without resurfaced patellae (116.4 ± 8.9 R, 121.5 ± 8.6 NR, *p* = 0.03, d = −0.58). Range of motion data are visualized in Figure 5.

### 3.4 Association Between Merchant Grade and PROMs

Spearman correlation analyses demonstrated no statistically significant association between baseline Merchant grade and postoperative PROMs within either cohort. (Table 4). In the non‐resurfacing cohort, Merchant grade was not significantly associated with PROMIS Physical Function (ρ = 0.09, *p* = 0.57), PROMIS Pain Interference (ρ = −0.02, *p* = 0.89), VAS pain (ρ = 0.13, *p* = 0.38), Forgotten Joint Score (ρ = −0.15, *p* = 0.33), KOOS total score (ρ = −0.18, *p* = 0.24), SMC total score (ρ = −0.11, *p* = 0.47), KSS knee score (ρ = −0.09, *p* = 0.57), or KSS function score (ρ = −0.04, *p* = 0.79). Similarly, in the resurfacing cohort, Merchant grade was not significantly associated with PROMIS Physical Function (ρ = −0.06, *p* = 0.69), PROMIS Pain Interference (ρ = 0.08, *p* = 0.61), VAS pain (ρ = 0.10, *p* = 0.50), Forgotten Joint Score (ρ = −0.12, *p* = 0.43), KOOS total score (ρ = −0.09, *p* = 0.55), SMC total score (ρ = −0.03, *p* = 0.83), KSS knee score (ρ = −0.07, *p* = 0.65), or KSS function score (ρ = −0.14, *p* = 0.35). These findings suggest that baseline radiographic patellofemoral arthritis severity was not significantly associated with 12‐month patient‐reported outcomes in either cohort.

**TABLE 4:** Association between baseline merchant grade and 12‐month PROMs.

Outcome measure	Non‐resurfaced spearman rho	Non‐resurfaced *p*‐value	Resurfaced spearman rho	Resurfaced *p*‐value
PROMIS physical function t‐score	0.09	0.57	−0.06	0.69
PROMIS pain interference t‐score	−0.02	0.89	0.08	0.61
Visual analog scale (VAS)	0.13	0.38	0.10	0.50
Forgotten joint score	−0.15	0.33	−0.12	0.43
KOOS total score	−0.18	0.24	−0.09	0.55
SMC PF pain score	−0.07	0.64	0.004	0.98
SMC PF function score	−0.17	0.26	−0.11	0.47
SMC PF total score	−0.11	0.47	−0.03	0.83
KSS knee score	−0.09	0.57	−0.07	0.65
KSS function score	−0.04	0.79	−0.14	0.35

*Note:* Spearman correlation analyses were performed within each cohort to assess associations between baseline Merchant grade and 12‐month postoperative patient‐reported outcome measures. No statistically significant correlations were observed.

### 3.5 Complications

Through 12 months of follow‐up, no extensor mechanism complications, including patellar fracture, patellar component loosening, or patellar maltracking, were observed in either cohort. There were no periprosthetic joint infections and no reoperations or revisions in either the resurfacing or non‐resurfacing group during the study period. No patients required readmission for a procedure‐related complication. As no complications or adverse events occurred in either group, no additional intervention or management was required, and complication rates did not differ between cohorts.

## 4 Discussion

In this retrospective cohort study of patients undergoing primary medial‐stabilized total knee arthroplasty (MS TKA) with a round‐on‐round, one‐to‐one congruent implant (Sphere), we found that patellar resurfacing was not associated with superior pain relief or improved patient‐reported outcomes at approximately 1 year postoperatively. Across our primary pain‐ and function‐centered endpoints, patients achieved comparable outcomes whether the patella was resurfaced or not. Although select secondary measures differed between groups (including small differences in knee flexion and certain KOOS subscales), these findings did not translate into a consistent, clinically meaningful advantage for either strategy. Collectively, our results support the concept that, in the setting of this MS implant design, both resurfacing and non‐resurfacing are reasonable approaches, and that routine resurfacing is unlikely to provide broad, measurable benefit at short‐term follow‐up.

### 4.1 Pain and Patient‐Reported Outcomes

Patellar resurfacing remains one of the most debated decisions in primary TKA, in part because prior evidence has been mixed and often implant‐dependent. While some studies and meta‐analyses in more traditional posterior‐stabilized or cruciate‐retaining designs suggest resurfacing can reduce anterior knee pain or certain patella‐related reoperations, other reports demonstrate minimal differences in PROMs and functional recovery [9, 10]. The present study adds implant‐specific evidence within the growing category of medial‐stabilized designs, where patellofemoral mechanics and kinematics may differ from other TKA constructs [16]. Our findings align with the growing body of literature suggesting that in modern, “patellar‐friendly” or kinematically oriented designs, routine resurfacing may not be necessary to achieve excellent short‐term pain and functional outcomes in most patients [11, 14].

From a patient‐centered perspective, the most important finding is the absence of meaningful differences in pain and overall function at one year. Postoperative pain, assessed by VAS and SMC pain, was similar between cohorts, and PROM domains capturing global function and symptom burden (e.g., PROMIS PF/PI, overall KOOS, KSS function) did not differ. These results suggest that for this MS implant, resurfacing does not reliably improve the outcome metrics most closely tied to patient satisfaction and daily performance. Clinically, this supports a selective resurfacing strategy rather than routine resurfacing, particularly when balanced against the additional procedure time and implant‐specific considerations.

### 4.2 Range of Motion and Secondary Outcomes

Exploratory analyses showed statistically significant but modest differences favoring the non‐resurfacing cohort in postoperative knee flexion and in two KOOS subscales (ADL and Symptoms). Knee flexion was slightly greater in the non‐resurfacing group at follow‐up, but the absolute difference was small and did not translate into consistent advantages across broader functional or satisfaction measures consistent with previous literature [17, 18]. By contrast, no between‐group differences were observed across the remaining KOOS domains (Pain, Sport/Rec, and Quality of Life), total KOOS, or other PROM instruments including PROMIS measures, the Forgotten Joint Score, the Knee Society Score, or the Single Assessment Numeric Evaluation. Taken together, the overall pattern suggests broadly comparable patient‐reported outcomes, with only isolated subscale differences. Given the large number of secondary comparisons performed, these findings should be interpreted cautiously as exploratory and hypothesis‐generating rather than definitive evidence of superiority.

### 4.3 Patellofemoral Arthritis Severity

Baseline Merchant grade was not significantly different between groups, and severe PF arthritis was not significantly overrepresented in either cohort. Additionally, baseline Merchant grade was not significantly associated with 12‐month PROMs within either the resurfaced or non‐resurfaced cohort. These findings reduce concern that differences in baseline radiographic PF arthritis severity substantially biased the observed short‐term outcome comparisons.

### 4.4 Perioperative Outcomes

With respect to perioperative outcomes, resurfacing was associated with longer operative and tourniquet times, which is expected given the additional procedural steps required to prepare and implant a patellar component. While these differences were statistically significant, their clinical significance is likely limited in most cases, particularly in centers with standardized perioperative pathways and low complication rates. Length of stay differed between groups by approximately several hours; however, this magnitude of difference is unlikely to represent a clinically meaningful change in recovery trajectory and may be influenced by discharge logistics and other nonoperative factors [19, 20]. Importantly, early postoperative opioid requirements (MME) and perioperative hemoglobin change were similar between groups, suggesting that patellar management in this setting did not meaningfully affect immediate postoperative pain control or short‐term physiologic recovery.

### 4.5 Complications

Complication profiles were reassuring in both cohorts at one year. We observed no extensor mechanism complications and no infections or reoperations for prosthetic joint infection. While these events are uncommon and our study is not powered to detect small differences in rare complications, the absence of patella‐related mechanical complications in either group supports the short‐term safety of both strategies when performed within appropriate surgical indications and technique [21].

### 4.6 Strengths and Limitations

This study has several strengths. The design isolated the effect of patellar management by comparing two high‐volume surgeons with consistent and opposite resurfacing practices using a single, uniform medial‐stabilized implant, thereby minimizing implant‐related and technique‐related variability. Outcomes were assessed prospectively at a standardized 1‐year follow‐up using a comprehensive panel of validated patient‐reported outcome measures, physical examination, and perioperative data. In addition, baseline patellofemoral arthritis severity was rigorously characterized using blinded, multi‐reviewer Merchant grading with substantial interrater reliability, allowing this potential confounder to be directly evaluated rather than assumed. Our study also has several limitations to consider. This was a retrospective, nonrandomized study in which patellar management was entirely surgeon dependent. Although baseline demographics and radiographic patellofemoral arthritis severity were not significantly different between groups, resurfacing status remained perfectly confounded by surgeon. Therefore, unmeasured surgeon‐level differences, subtle technical variation, referral patterns, or discharge practices may still have influenced perioperative and clinical outcomes. Although Merchant grading provided a standardized radiographic measure of PF arthritis severity, radiographic severity may not fully capture cartilage status, dynamic patellofemoral tracking, or symptom burden. The number of patients with severe PF arthritis was limited, and no patients were classified as Merchant grade 4; therefore, subgroup analyses in end‐stage patellofemoral disease remain underpowered. Finally, the study was conducted at a single tertiary care center using one MS implant design, which may limit generalizability.

## 5 Conclusion

In this retrospective cohort of primary medial‐stabilized TKA using the Sphere implant, patellar resurfacing was not associated with superior short‐term pain relief or patient‐reported functional outcomes at approximately one year. Additionally, patellofemoral arthritis severity was not significantly imbalanced between groups and was not significantly associated with postoperative PROMs within either cohort. These findings suggest that resurfacing did not provide a consistent short‐term clinical advantage in this MS implant cohort. Larger prospective studies with greater representation of severe patellofemoral arthritis are needed to determine whether specific high‐severity subgroups benefit from resurfacing.

## Author Contributions

**T. Jacob Selph Jr:** formal analysis, visualization, writing – original draft, writing – review and editing, data curation. **Isaac Sontag‐Milobsky:** conceptualization, data curation, investigation, writing – original draft, writing – review and editing, project administration. **Kevin D. Hardt:** conceptualization, methodology, visualization, writing – review and editing. **Giancarlo Riccobono:** formal analysis, writing – review and editing, writing – original draft, data curation. **David W. Manning:** conceptualization, supervision, project administration, methodology, writing – review and editing.

## Funding

The authors received no financial support for the research, authorship, or publication of this article.

## Ethics Statement

This study was conducted in accordance with the principles of the Declaration of Helsinki and received approval from the Institutional Review Board (IRB). Written informed consent was obtained from all study participants prior to their inclusion in the study.

## Conflicts of Interest

The authors declare no conflicts of interest.

## Data Availability

Research data are not shared.
